# Global burden and cross-country inequalities in gallbladder and biliary tract cancer (1990–2021) with projections to 2050: insights from the global burden of disease study 2021

**DOI:** 10.3389/fmed.2025.1520714

**Published:** 2025-05-12

**Authors:** Diya Xie, Fengmin Liu, Daosen Zhou, Qiang Zhu, Fangting Xiao, Kun Zhang

**Affiliations:** ^1^Department of General Surgery, Fuzhou First General Hospital Affiliated with Fujian Medical University, Fuzhou, Fujian, China; ^2^Department of Endocrinology, Fuzhou First General Hospital Affiliated with Fujian Medical University, Fuzhou, Fujian, China; ^3^Department of Breast Surgery, Fuzhou First General Hospital Affiliated with Fujian Medical University, Fuzhou, Fujian, China

**Keywords:** Bayesian age-period-cohort, global burden of disease, gallbladder and biliary tract cancer, health inequality, joinpoint model, unexpected disparities

## Abstract

**Background:**

Gallbladder and biliary tract cancer (GBTC) presents a worldwide health challenge with a poor prognosis. Previous studies indicated an escalating burden and potential health inequalities, necessitating an updated investigation.

**Methods:**

This study utilized data from the Global Burden of Disease (GBD) study, covering 204 countries from 1990 to 2021. Joinpoint regression evaluated temporal trends in age-standardized incidence rates (ASIR) and age-standardized disability-adjusted life years rates (ASDR) for GBTC. The Bayesian age-period-cohort (BAPC) model projected disease burden up to 2050. Inequality analysis assessed disparities by genders across countries, and decomposition analysis determined the contributions of demographic and epidemiological factors.

**Results:**

From 1990 to 2021, the incident cases of GBTC increased from 107,797 to 216,768, while Disability-Adjusted Life Years (DALYs) rose from 2,326,089 years to 3,732,121. Joinpoint regression analysis revealed a global decrease in ASIR (AAPC = −0.39, 95% CI: −0.49 to −0.28) and ASDR (AAPC = −0.97, 95% CI: −1.07 to −0.88). Gender disparities were notable, with a polar reversal observed: females exhibited consistently higher ASDR levels across three decades, although both ASDR and ASIR showed continuous decreases. In contrast, males experienced a decreased ASDR but increased ASIR, with both metrics eventually surpassing those of females. The projection model also suggested diverging ASIR trends between genders. Cross-country inequality analysis revealed persistent disparities, where higher SDI countries continue to bear a greater burden, and global improvement in health equity for males remains insufficient. Decomposition analysis indicated that population growth and ageing were primary drivers of disease burden increase, whereas epidemiological changes contributed to a reduction, particularly in higher SDI quintiles.

**Conclusion:**

Despite improvements, GBTC burden is still greater in high SDI regions compared to lower SDI areas, contrary to expectations. Unexpected polar reversal of gender differences warrants further attention.

## Introduction

Gallbladder and biliary tract cancers (GBTC) are relatively rare globally (1), but both exhibit a very poor prognosis (2, 3). The mean survival rate for patients with gallbladder cancer is approximately 6 months, with a five-year survival rate of only 5% (4). Patients with cholangiocarcinoma are frequently diagnosed at an advanced stage, with the median survival time for unresectable cases ranging from approximately 3 to 6 months (5). In recent years, advancement has been made in the diagnosis and treatment (6) of GBTC; however, understanding its etiology, risk factors, and treatment strategies remains a key focus in epidemiological research. Epidemiological studies have shown significant regional variations in the incidence of GBTC, with higher rates in certain geographical regions (7).

According to global studies based on the Global Burden of Disease (GBD), by 2019, the incidence rates of GBTC had increased by 1.85 times, mortality rates had escalated by 1.92 times, and disability-adjusted life years (DALY) had risen by 1.68 times compared to the year of 1990 (8). Previous research has leveraged the Quality of Care Index (QCI) to reveal substantial variations in healthcare quality across different regions and countries, highlighting disparities in the management and outcomes of GBTC (9). Despite a general decline, age-standardized incidence rates (ASIR) were continuously higher in females across most regions, particularly in Southeast Asia (10). However, in China, a contrasting trend was observed, with males exhibiting higher ASIR and age-standardized DALYs rates (ASDR) than females in 2019, and these rates have been increasing at a much faster pace since 1990 (11). While past studies have indicated health inequalities in the burden of GBTC in terms of region and gender (12), no study has quantitatively assessed the extent of this inequality. Furthermore, the composition factors underlying the changes in disease burden have not been thoroughly explored.

Addressing these gaps, this study builds upon previous research through 2019 and extends the analysis to 2021, investigating the disparities in the burden of GBTC across Socio-demographic Index (SDI) quintiles and genders. The joinpoint model was utilized to identify turning points in GBTC trends, and the Bayesian age-period-cohort (BAPC) model was employed to project future disease burden up to the year 2050. Additionally, the slope of inequality index (SII) and concentration index (CI) were used to quantify inequality in GBTC burden, providing a framework to assess disparities in incidence and DALY rates among different socio-demographic and gender groups. This study aims to provide a comprehensive understanding of the inequalities in GBTC burden and to explore the contributing factors to these disparities, offering valuable insights for healthcare policy and resource allocation.

## Methods

### Data resources

This study leveraged data from the Global Burden of Disease (GBD) study, which meticulously collected data on disease burden, encompassing major diseases, injuries, and risk factors across 204 countries and territories, spanning 1990 to 2021 (13). The GBD study employs rigorous methodologies to ensure data accuracy and completeness, including the use of DisMod-MR 2.1 for disease modeling, geographical restrictions and assumption methods for missing data, garbage code handling for cause-of-death analyses, and supplementary data sources and covariates to enhance data comprehensiveness and reliability (14). Complied Guidelines for Accurate and Transparent Health Estimates Reporting (GATHER) (15) statement, the methods employed in the GBD 2021 study ensure rigour and transparency in elucidating the burden and dynamics of GBTC on a global range. Owing to the low prevalence and unfavourable prognosis, incidence and DALYs were chosen as the principal metrics for assessing the burden of GBTC. All relative data were accessible through the Global Health Data Exchange (16). The methodology used to calculate the above measures in this study had been previously documented (17). For precise disease identification of GBTC, all cancers were coded within C23 to C24.9 according to the 10th version of the International Classification of Diseases (ICD-10). Intrahepatic bile duct cancer (C22.1) was not included in GBTC by GBD guidelines.

### Socio-demographic index (SDI)

The SDI serves as a composite indicator of a country or region’s development status, based on factors including per capita income, average educational attainment of individuals aged 15 and older, and the total fertility rate among women younger than 25 (18). For this study, countries were stratified into five SDI quintiles (low, low-middle, middle, high-middle, and high) to assess the impact of socio-demographic development on the burden of GBTC. This stratification allowed us to identify disparities in GBTC incidence and DALYs across different levels of development. The SDI quintiles provide a framework for understanding how socio-demographic factors influence the epidemiology of GBTC globally.

### Joinpoint regression analysis

Joinpoint regression analysis software (19) (v4.9.1.0) was employed as a pivotal method to investigate temporal trends in the age-standardized DALYs rate (ASDR) and age-standardized incidence rate (ASIR) of GBTC from 1990 to 2021. Joinpoint itself does not directly eliminate all confounding effects. However, by standardizing data for age and other demographic factors, as well as conducting stratified/segmented analyses, it can better reduce the impact of external interfering factors, thereby improving the reliability of trend analysis results. This analytical approach is particularly adept at identifying points in time, or “joinpoints,” where a notable linear trend shifts. It effectively captures changes in incidence and DALY rates by annual percent change (APC) for each period (a linear trend on a log scale indicates a constant APC) and the average annual percentage change (AAPC), providing insights into the overall trajectory of GBTC burden during the 31 years. APC represents the average annual percentage change within each specific interval, while AAPC reflects the overall average annual change across the entire period. A number of +0.5 in this context signifies a 0.5% percent annual growth in the rate across the period.

### Projection analysis

The incidence and DALYs for the burden of GBTC from 2022 to 2050 were projected using the BAPC model, which allows for the analysis of how age, period, and cohort effects influence cancer rates. The nested Laplace approximations facilitate efficient computation of the posterior distributions, enabling the model to generate probabilistic projections without resorting to Markov chain Monte Carlo (MCMC) methods (20), thus avoiding complex convergence issues. The BAPC model is widely utilized for analyzing and projecting age-specific cancer mortality and incidence rates, particularly in the context of significant demographic shifts. This model relies on the R packages BAPC and INLA for implementation, which enable the computation of well-calibrated probabilistic projections with relatively narrow uncertainty intervals (21). By extrapolating historical trends in cancer incidence and mortality rates, the BAPC model projects data up to the year 2050 while accounting for demographic changes such as shifts in population age structure, fertility rates, and mortality rates. These projections are relied on population forecasts from sources like the Institute for Health Metrics and Evaluation (IHME), which provide detailed projections of population dynamics extending to the year 2100 (22). These demographic projections are integrated into the BAPC model to ensure that the cancer burden projections reflect realistic future population scenarios. Given that the BAPC model assumes the continuation of historical trends, its interpretive significance lies more in the potential consequences if the current status is maintained, serving as a lesson from the past. An optimal condition for the BAPC model is that the data should conform to a Poisson distribution; hence, while this study have generated predictions for DALYs, these should be considered exploratory. In contrast, the predictions of incidence hold greater referential significance.

### Decomposition analysis

Decomposition analysis (23) based on the methodology of Gupta (24) was applied to dissected the factors contributing to the observed changes in GBTC burden from 1990 to 2021. The analysis scrutinized population structures and epidemiological shifts, unveiling their impacts by calculating the proportion of change. The percentage changes were calculated relative to the figures from 1990 as a baseline, thereby enhancing the comparability of the results across different SDI quintiles or gender groups within regions.

### Cross-country inequality analysis

To assess disparities in the burden of GBTC across nations, we utilized the SII and CI (25, 26). These indices unveiled absolute and relative sociodemographic-related discrepancies, respectively. The SII was derived by regressing a measure (DALYs or incidence) against a socio-demographic development scale, representing the midpoint of the cumulative value of the population ranked by SDI. The CI for health inequality was calculated by fitting a Lorenz concentration curve to the cumulative measure and the cumulative distribution of populations ranked by SDI. This was followed by numerical integration to determine the area under the curve; a lower absolute value of CI indicates more equal distribution of health in that area. Both indices can be calculated following the guidelines outlined in the World Health Organization’s Handbook on Health Inequality Monitoring (26).

### Statistics analysis

We performed all data collection, statistical analysis, and visualization with R software (v4.3.1) using *MASS, ggplot2*, and *gt*. The significance of estimated values was assessed by the Wald chi-squared test. Age-standardized rates were reported per 100,000 population, with their respective 95% uncertainty intervals (95% UI). Statistics calculated by models were reported by the 95% confidential intervals (95% CI), and the statistical significance was considered at a pval threshold of < 0.05. Given the large volume of data and space constraints, we used a streamlined approach: when the 95% CI of a parameter estimate does not include zero, it is interpreted as statistically significant (*p* < 0.05). Detailed materials on these analyses, including age-standardized rates, SDI, joinpoint regression, and decomposition methods, are provided in the Supplementary methods.

## Result

### Global trends and burden of GBTC

Globally, the number of DALYs of GBTC increased from 2,326,089 years (95% UI: 2,054,497 to 2,582,958) in 1990 to 3,732,121 (95% UI: 3,102,893 to 4,316,985) in 2021 (Table 1, (Supplementary Table S1), while incidence cases rose from 107,797 (95% UI, 96,890 to 117,511) in 1990 to 216,768 (95% UI, 181,888 to 245,237) in 2021 (Table 2 and Supplementary Table S2).

**Table 1:** DALYs for GBTC: age-standardized rates with 95% UI and AAPC with 95% CI, 1990–2021.

Category	GBTC burden in 1990		GBTC burden in 2021		GBTC burden from 1990–2021
	DALYs No. *10^3^ (95% UI)	ASDR per 100,000 No. (95% UI)	DALYs No. *10^3^ (95% UI)	ASDR per 100,000 No.(95% UI)	AAPC No. (95% CI)
Global	2326.09 [2054.50 to 2582.96]	58.58 [52.21 to 64.88]	3732.12 [3102.89 to 4316.99]	43.20 [36.01 to 49.88]	−0.97 [−1.07 to −0.88]
Female	1414.96 [1232.93 to 1649.43]	66.31 [57.94 to 77.19]	2047.32 [1624.44 to 2400.74]	44.55 [35.35 to 52.31]	−1.27 [−1.34 to −1.20]
Male	911.13 [740.61 to 1080.56]	49.55 [40.88 to 58.55]	1684.80 [1229.53 to 1996.60]	41.95 [30.95 to 49.56]	−0.53 [−0.63 to −0.43]
High SDI	840.67 [781.77 to 877.67]	76.34 [70.96 to 79.76]	910.14 [801.57 to 991.39]	43.16 [38.51 to 46.77]	−1.83 [−1.97 to −1.70]
Female	508.59 [472.32 to 534.00]	80.88 [74.96 to 84.80]	456.22 [391.28 to 504.57]	39.20 [34.11 to 42.80]	−2.34 [−2.46 to −2.21]
Male	332.09 [303.02 to 351.26]	70.45 [64.47 to 74.44]	453.91 [392.57 to 498.66]	47.85 [41.22 to 52.52]	−1.25 [−1.33 to −1.16]
High middle SDI	627.56 [533.88 to 680.18]	62.71 [53.60 to 67.86]	855.46 [651.94 to 983.36]	43.27 [32.90 to 49.76]	−1.22 [−1.42 to −1.02]
Female	383.39 [326.44 to 420.43]	68.18 [58.04 to 74.77]	444.68 [342.50 to 524.57]	41.17 [31.65 to 48.58]	−1.63 [−1.82 to −1.43]
Male	244.18 [184.83 to 282.65]	55.78 [42.75 to 64.13]	410.78 [272.72 to 513.90]	45.87 [30.63 to 57.07]	−0.71 [−0.82 to −0.60]
Middle SDI	533.71 [457.80 to 673.94]	49.77 [43.05 to 63.31]	1116.08 [907.04 to 1399.23]	40.66 [32.88 to 51.04]	−0.64 [−0.74 to −0.55]
Female	313.38 [253.28 to 410.89]	57.19 [46.67 to 75.02]	593.64 [469.01 to 754.26]	41.34 [32.63 to 52.48]	−1.03 [−1.17 to −0.90]
Male	220.33 [167.46 to 322.67]	41.98 [32.32 to 61.70]	522.44 [356.02 to 690.40]	40.03 [27.50 to 52.95]	−0.14 [−0.26 to −0.03]
Low middle SDI	255.59 [215.07 to 361.91]	39.66 [33.71 to 56.29]	681.87 [529.87 to 840.52]	45.87 [35.74 to 56.91]	0.49 [0.37 to 0.62]
Female	163.91 [131.17 to 250.63]	51.43 [41.57 to 79.26]	439.82 [315.64 to 592.01]	56.83 [40.94 to 76.54]	0.34 [0.16 to 0.52]
Male	91.68 [68.64 to 130.42]	28.24 [21.31 to 40.33]	242.06 [169.47 to 309.75]	34.12 [23.84 to 43.74]	0.61 [0.44 to 0.79]
Low SDI	65.25 [50.72 to 90.20]	27.06 [21.30 to 37.66]	165.54 [115.08 to 207.61]	31.49 [21.79 to 39.14]	0.52 [0.35 to 0.68]
Female	43.44 [31.12 to 66.04]	36.39 [26.61 to 55.42]	111.26 [72.92 to 145.96]	41.18 [27.01 to 53.80]	0.43 [0.21 to 0.64]
Male	21.81 [14.92 to 30.39]	18.07 [12.41 to 25.20]	54.28 [31.35 to 70.65]	21.39 [12.53 to 27.72]	0.56 [0.38 to 0.75]

DALYs, disability-adjusted life years; ASDR, age standardized disability-adjusted life years rate; AAPC, average annual percent change; SDI, Socio-Demographic Index; UI, uncertainty interval; 95% CI, 95% confidential interval. The symbol *10^3^ indicates the value multiplied by 1000.

**Table 2:** Incidence for GBTC: age-standardized rates with 95% UI and AAPC with 95% CI, 1990–2021.

Category	GBTC burden in 1990		GBTC burden in 2021		GBTC burden from 1990–2021
	Incidence cases No. *10^3^ (95% UI)	ASIR per 100,000 No. (95% UI)	Incidence cases No. *10^3^ (95% UI)	ASIR per 100,000 No.(95% UI)	AAPC No. (95% CI)
Global	107.80 [96.89 to 117.51]	2.89 [2.59 to 3.15]	216.77 [181.89 to 245.24]	2.56 [2.16 to 2.89]	−0.39 [−0.49 to −0.28]
Female	66.88 [59.47 to 75.64]	3.22 [2.85 to 3.64]	115.98 [94.59 to 132.85]	2.50 [2.03 to 2.86]	−0.82 [−0.94 to −0.69]
Male	40.92 [34.82 to 46.84]	2.46 [2.13 to 2.80]	100.78 [77.37 to 116.40]	2.65 [2.06 to 3.04]	0.23 [0.08 to 0.38]
High SDI	49.14 [45.63 to 51.24]	4.38 [4.06 to 4.57]	78.92 [69.10 to 86.39]	3.50 [3.11 to 3.80]	−0.74 [−0.91 to −0.58]
Female	30.51 [27.97 to 32.10]	4.58 [4.21 to 4.80]	40.38 [33.43 to 45.18]	3.12 [2.68 to 3.44]	−1.26 [−1.35 to −1.18]
Male	18.63 [17.48 to 19.39]	4.09 [3.82 to 4.26]	38.54 [34.26 to 42.00]	3.96 [3.51 to 4.31]	−0.14 [−0.27 to −0.00]
High middle SDI	27.57 [23.97 to 29.65]	2.88 [2.50 to 3.09]	52.99 [40.58 to 61.16]	2.68 [2.05 to 3.09]	−0.32 [−0.68 to 0.03]
Female	17.34 [15.15 to 18.77]	3.11 [2.71 to 3.37]	27.53 [21.12 to 32.39]	2.48 [1.90 to 2.92]	−0.72 [−1.03 to −0.40]
Male	10.23 [8.03 to 11.64]	2.57 [2.04 to 2.88]	25.46 [17.12 to 31.89]	2.94 [1.99 to 3.65]	0.44 [0.20 to 0.68]
Middle SDI	19.72 [17.10 to 25.13]	2.02 [1.77 to 2.57]	52.74 [42.75 to 68.35]	2.00 [1.63 to 2.60]	−0.02 [−0.14 to 0.10]
Female	11.72 [9.62 to 15.43]	2.30 [1.91 to 3.01]	27.34 [21.43 to 35.03]	1.95 [1.53 to 2.51]	−0.51 [−0.62 to −0.39]
Male	8.00 [6.17 to 11.62]	1.72 [1.34 to 2.47]	25.40 [17.60 to 34.43]	2.07 [1.45 to 2.80]	0.61 [0.50 to 0.73]
Low middle SDI	8.97 [7.69 to 12.69]	1.53 [1.31 to 2.18]	25.88 [20.36 to 32.12]	1.86 [1.47 to 2.31]	0.66 [0.50 to 0.82]
Female	5.73 [4.64 to 8.73]	1.96 [1.58 to 2.99]	16.58 [12.14 to 22.29]	2.26 [1.67 to 3.03]	0.48 [0.29 to 0.66]
Male	3.24 [2.46 to 4.62]	1.10 [0.84 to 1.57]	9.30 [6.50 to 11.98]	1.42 [0.98 to 1.82]	0.84 [0.79 to 0.90]
Low SDI	2.25 [1.77 to 3.12]	1.03 [0.82 to 1.43]	6.08 [4.21 to 7.57]	1.28 [0.88 to 1.57]	0.75 [0.55 to 0.95]
Female	1.49 [1.09 to 2.26]	1.36 [0.99 to 2.07]	4.06 [2.65 to 5.30]	1.65 [1.07 to 2.13]	0.66 [0.42 to 0.89]
Male	0.76 [0.53 to 1.06]	0.70 [0.48 to 0.97]	2.02 [1.18 to 2.61]	0.89 [0.53 to 1.14]	0.80 [0.55 to 1.05]

ASIR, age standardized incidence rate; AAPC, average annual percent change; SDI, Socio-Demographic Index; UI, uncertainty interval; 95% CI, 95% confidential interval. The symbol *10^3^ indicates the value multiplied by 1000.

### Statistical inference of results

**Figure 1:**
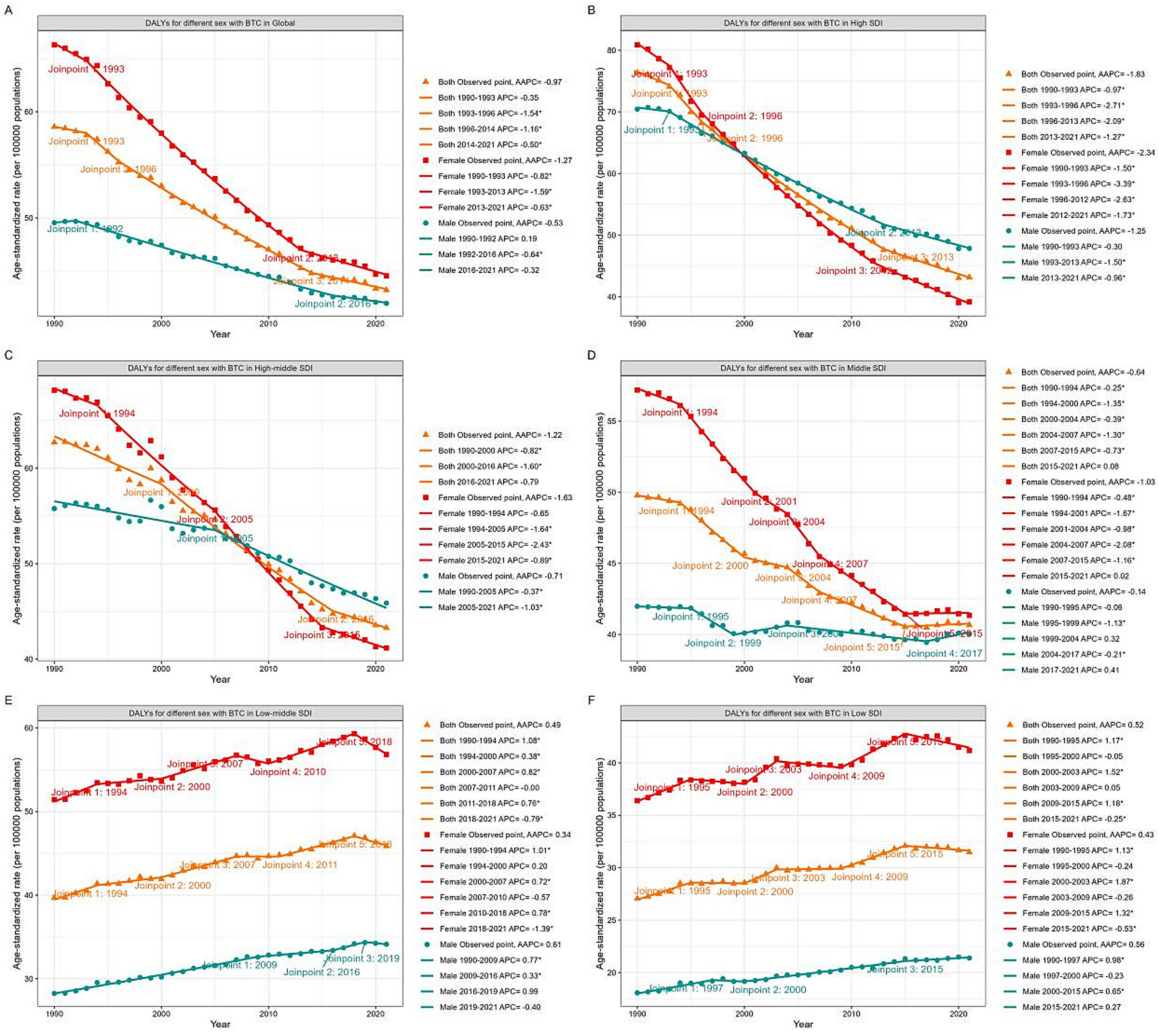
Global trends in ASDR for GBTC from 1990 to 2021. ASDR for GBTC in whole population and by genders in Global **(A)**, High SDI quintile **(B)**, High-middle SDI quintile **(C)**, Middle SDI quintile **(D)**, Low-middle SDI quintile **(E)**, and Low SDI quintile **(F)**. ASDR, age standardized disability-adjusted life-years rate; GBTC, gallbladder and biliary tract cancer; AAPC, average annual percent change; APC, annual percent change; SDI, Socio-Demographic Index.

**Figure 2:**
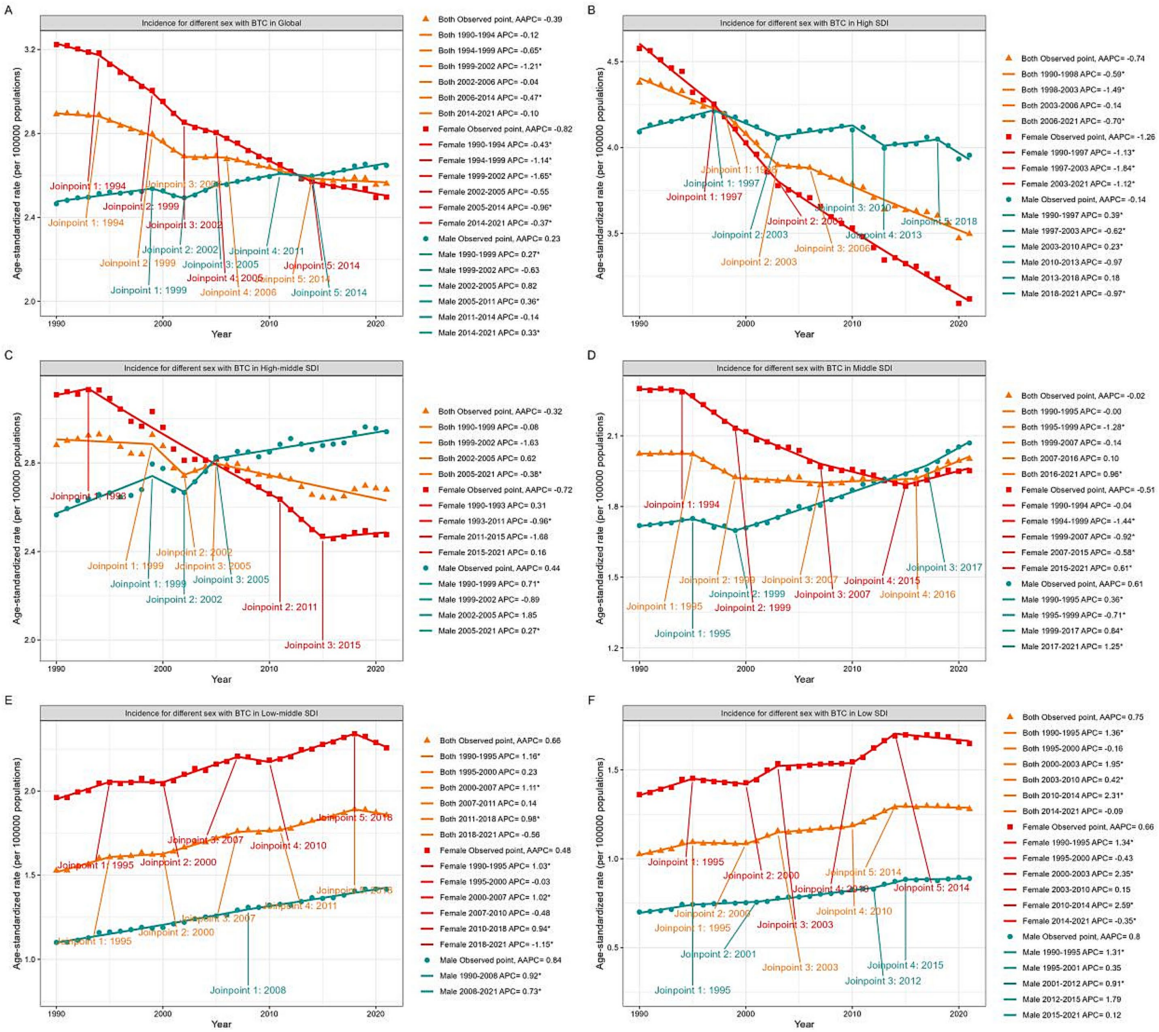
Global trends in ASIR for GBTC from 1990 to 2021. ASIR for GBTC in whole population and by genders in Global **(A)**, High SDI quintile **(B)**, High-middle SDI quintile **(C)**, Middle SDI quintile **(D)**, Low-middle SDI quintile **(E)**, and Low SDI quintile **(F)**. ASIR, age standardized incidence rate; GBTC, gallbladder and biliary tract cancer; AAPC, average annual percent change; APC, annual percent change; SDI, Socio-Demographic Index.

The joinpoint regression analysis identified significant changes in the trends of ASIR and ASDR over the study period. Joinpoint regression analysis revealed significant decreasing trends for both the ASDR (AAPC = −0.97%; 95% CI: −1.07 to −0.88) and ASIR (AAPC = −0.39%; 95% CI: −0.49 to −0.28) from 1990 to 2021. For females, both ASDR (Figure 1) and ASIR (Figure 2) continuously decreased, with AAPCs of −1.27% (95% CI: −1.34 to −1.20) and −0.82% (95% CI: −0.94 to −0.70), respectively. In contrast, while ASDR for males also decreased (AAPC = −0.53%; 95% CI: −0.63 to −0.43), the ASIR increased (AAPC = 0.23%; 95% CI: 0.08 to 0.38), remaining stable during the periods 1999–2005 and 2011–2014. Notably, the ASDR levels kept continuously higher in females than males across the three decades, whereas the ASIR levels in males surpassed those in females after 2013.

### Analysis by SDI quintiles

Analysis of regions stratified by five SDI quintiles revealed distinct trends in ASDR (Figure 1, Supplementary Figure S1). The high SDI quintile exhibited the most significant decrease in ASDR (AAPC = −1.83%; 95% CI: −1.97 to −1.70), followed by the high-middle (AAPC = −1.22%; 95% CI: −1.42 to −1.02) and middle (AAPC = −0.64%; 95% CI: −0.74 to −0.55) quintiles. Conversely, the low-middle (AAPC = 0.49%; 95% CI: 0.37 to 0.62) and low (AAPC = 0.52%; 95% CI: 0.35 to 0.68) SDI quintiles showed increasing ASDR trends. Notably, the highest ASDR shifted from the high SDI quintile in 1990 to the low-middle SDI quintile by 2021.

Significant gender disparities were observed across different SDI quintiles. In the high, high-middle, and middle SDI quintiles, both genders exhibited decreasing ASDR trends, with males experiencing a slower decline. In the high SDI quintile, male ASDR values exceeded those of females after 2000, and in the high-middle SDI quintile, this occurred after 2007. Within the middle SDI quintile, ASDR stabilized for females (2015–2021) and males (2017–2021) in recent years. Conversely, in the low-middle and low SDI quintiles, ASDR for both genders increased over the three decades, with females consistently maintaining higher rates than males.

The ASIR exhibited similar patterns across SDI quintiles (Figure 2, Supplementary Figure S2). The high SDI quintile exhibited the most significant decline in ASIR (AAPC = −0.74%; 95% CI: −0.91 to −0.58), while the high-middle and middle SDI quintile remained stable (95% CI contained zero). The low-middle (AAPC = 0.66%; 95% CI: 0.50 to 0.82) and low (AAPC = 0.75%; 95% CI: 0.56 to 0.95) SDI quintiles both demonstrated increasing trends in ASIR. In the high SDI quintile, ASIR declined for both genders, with males showing a significantly lower slope. Male ASIR surpassed female rates after 1997. In the high-middle and middle SDI quintiles, male ASIR continued to increase, while female ASIR generally decreased but showed an upward trend after 2015. Notably, male ASIR surpassed females after 2005 in the high-middle SDI quintile and after 2013 in the middle SDI quintile. In the low and low-middle SDI quintiles, ASIR for both genders increased over the past three decades, with females consistently maintaining higher rates than males.

### Country-level analysis

**Figure 3:**
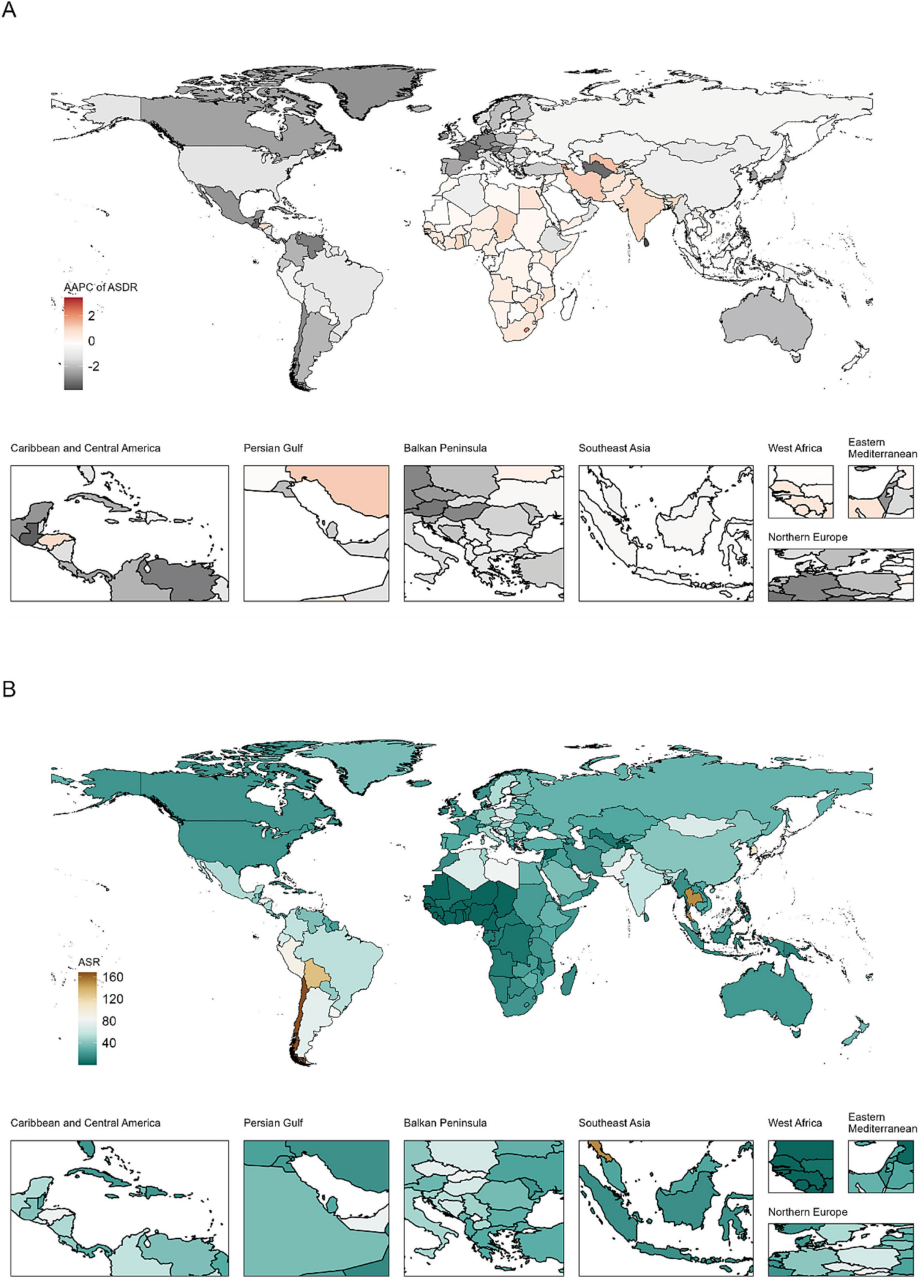
AAPC in ASDR of GBTC from 1990 to 2021 **(A)**, and ASDR values for GBTC in 204 Countries and Territories in 2021 **(B)**. ASDR, age standardized disability-adjusted life-years rate; GBTC, gallbladder and biliary tract cancer; AAPC, average annual percent change.

Among individual countries and territories (Figure 3, Supplementary Figure S3; Supplementary Tables S1, S2), Chile and Bolivia consistently topped the ASDR rankings in 1990 and 2021. Korea, previously in the second position in 1990, was overtaken by Thailand in 2021. Regarding ASIR, Chile and Korea maintained their lead, while Japan, third in 1990, was replaced by Thailand in 2021. Notably, Chile, despite having the highest ASIR and ASDR values from 1990 to 2021, exhibited a significant declining trend, with AAPCs of −2.08% (95% CI: −2.46 to −1.70) for ASIR and −2.87% (95% CI: −3.24 to −2.49) for ASDR. Cabo Verde, Armenia, and Lesotho experienced the most rapid increases in both ASIR and ASDR.

### Projections up to 2050

**Figure 4:**
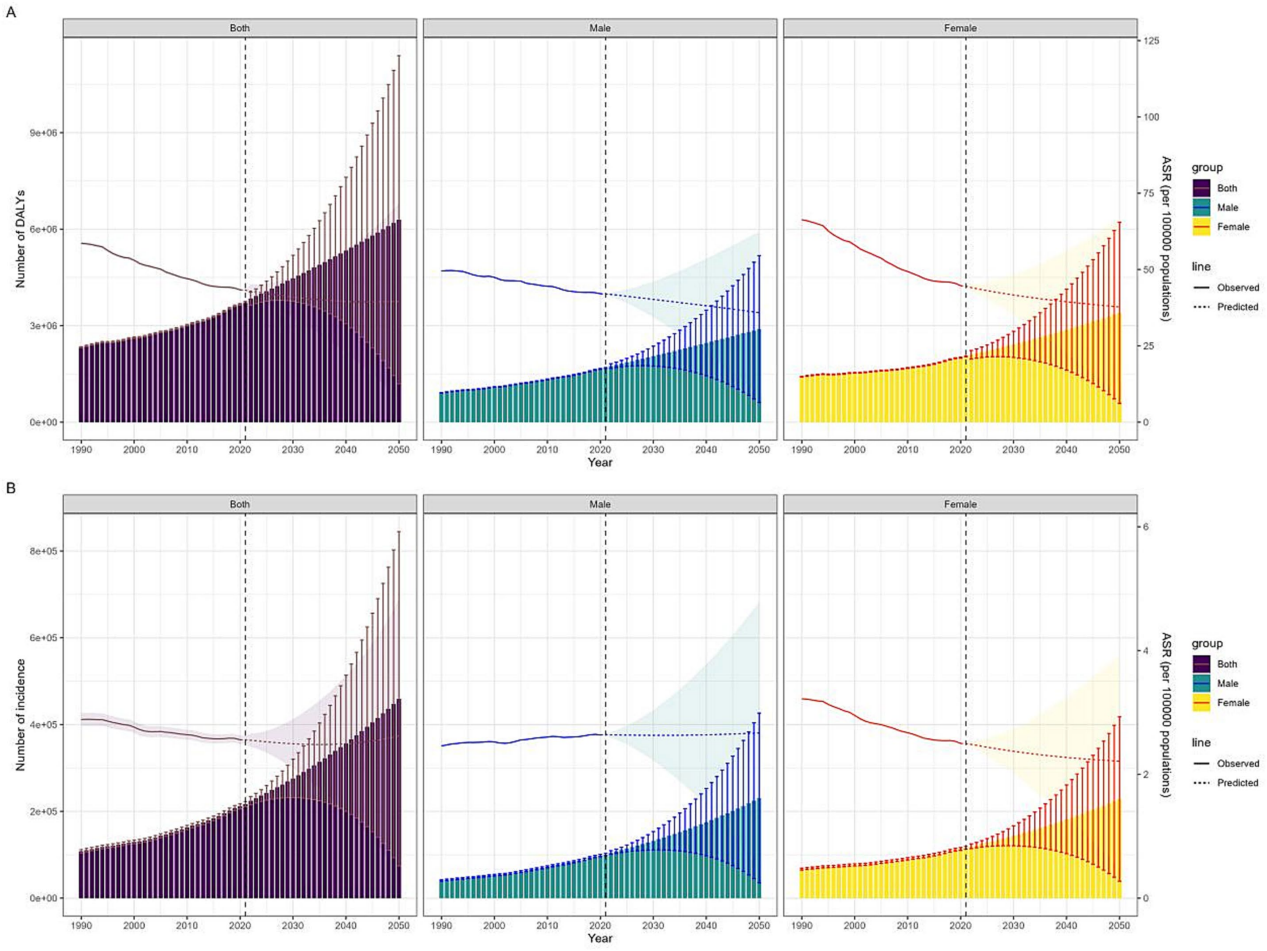
Trends in DALYs **(A)** and incidence **(B)** of GBTC (1990–2021 observed, 2022–2050 projected) by gender. The fan-shaped transparent area indicates 95% uncertainty intervals. ASR, age standardized rate; GBTC, gallbladder and biliary tract cancer; 95% UI, 95% uncertainty interval.

The BAPC model (Figure 4, Supplementary Table S4) predicts a 68.61% rise in global DALYs (Supplementary Table S3) from 2021 to 2050, reaching 6,295,282 years (95%UI: 1,181,195 to 11,412,032). Concurrently, the global ASDR is forecast to decrease by 8.40%, from 43.20 in 2021 (95%UI: 36.01 to 49.88) to 39.57 in 2050 (95%UI: 7.40 to 71.74). The ASDR for females and males is expected to decline to 37.73 (95%UI: 8.70 to 66.79) and 35.84 (95%UI: 9.30 to 62.35) by 2050, respectively. Additionally, the overall BTC incidence (Supplementary Table S4) is projected to rise by 111.86% to 459,247 cases in 2050 (95%UI: 74,364 to 844,403), with a 2.3% increase in ASIR compared to 2021. Gender-specific trends in ASIR are noted, with the female rate decreasing to 2.21 (95%UI: 0.50 to 3.93) and the male rate increasing to 2.67 (95%UI: 0.56 to 4.78) by 2050.

### Cross-country disparities of GBTC burden

A health inequality analysis was conducted to quantify the global disparities in GBTC burden across countries. In the global population, the slope index of inequality (SII) (Table 3) for GBTC incidence in 2021 was 2.76 (95% CI: 2.34 to 3.18), indicating an excess incidence of 2.76 (per 100,000 population) in the country with the highest SDI compared to the country with the lowest SDI in 1990. This gap further widened to 3.47 (95% CI: 2.92 to 4.01) in 2021. Conversely, the SII for crude DALYs decreased from 51.40 (95% CI: 42.94 to 59.86) in 1990 to 45.18 (95% CI: 36.37 to 53.98) in 2021. Among females, the SII trends for DALYs and incidence paralleled those observed in the overall population. In contrast, the SII for males increased for both DALYs and incidence. Over the 31-year period, a declining trend in the SII of the GBTC burden, encompassing both DALYs and incidence across genders, was observed in lower SDI quintiles, whereas higher quintiles exhibited an increasing trend (Supplementary Table S5).

**Table 3:** Global summary of SDI-related inequalities in GBTC DALYs and incidence for the total population and by gender.

Gender	Health inequality metrics	Year	DALYs		Incidence	
			Value	95% CI	Value	95% CI
Both	SII	1990	51.4	42.94 to 59.86	2.76	2.34 to 3.18
		2021	45.18	36.37 to 53.98	3.47	2.92 to 4.01
	CI	1990	0.34	0.28 to 0.41	0.45	0.38 to 0.52
		2021	0.23	0.17 to 0.29	0.39	0.31 to 0.47
Female	SII	1990	62.45	51.33 to 73.57	3.45	2.89 to 4.01
		2021	45.89	35.84 to 55.94	3.64	3.00 to 4.27
	CI	1990	0.34	0.28 to 0.41	0.46	0.39 to 0.53
		2021	0.18	0.12 to 0.23	0.35	0.28 to 0.42
Male	SII	1990	39.37	33.62 to 45.13	2.08	1.77 to 2.38
		2021	43.49	36.56 to 50.43	3.12	2.70 to 3.55
	CI	1990	0.34	0.27 to 0.40	0.44	0.37 to 0.51
		2021	0.29	0.21 to 0.36	0.43	0.34 to 0.52

SDI, Sociodemographic Index; DALYs, disability-adjusted life years; GBTC, gallbladder and biliary tract cancers; SII, Slope index of inequality; CI, Concentration index; 95% CI, 95% confidential interval.

**Figure 5:**
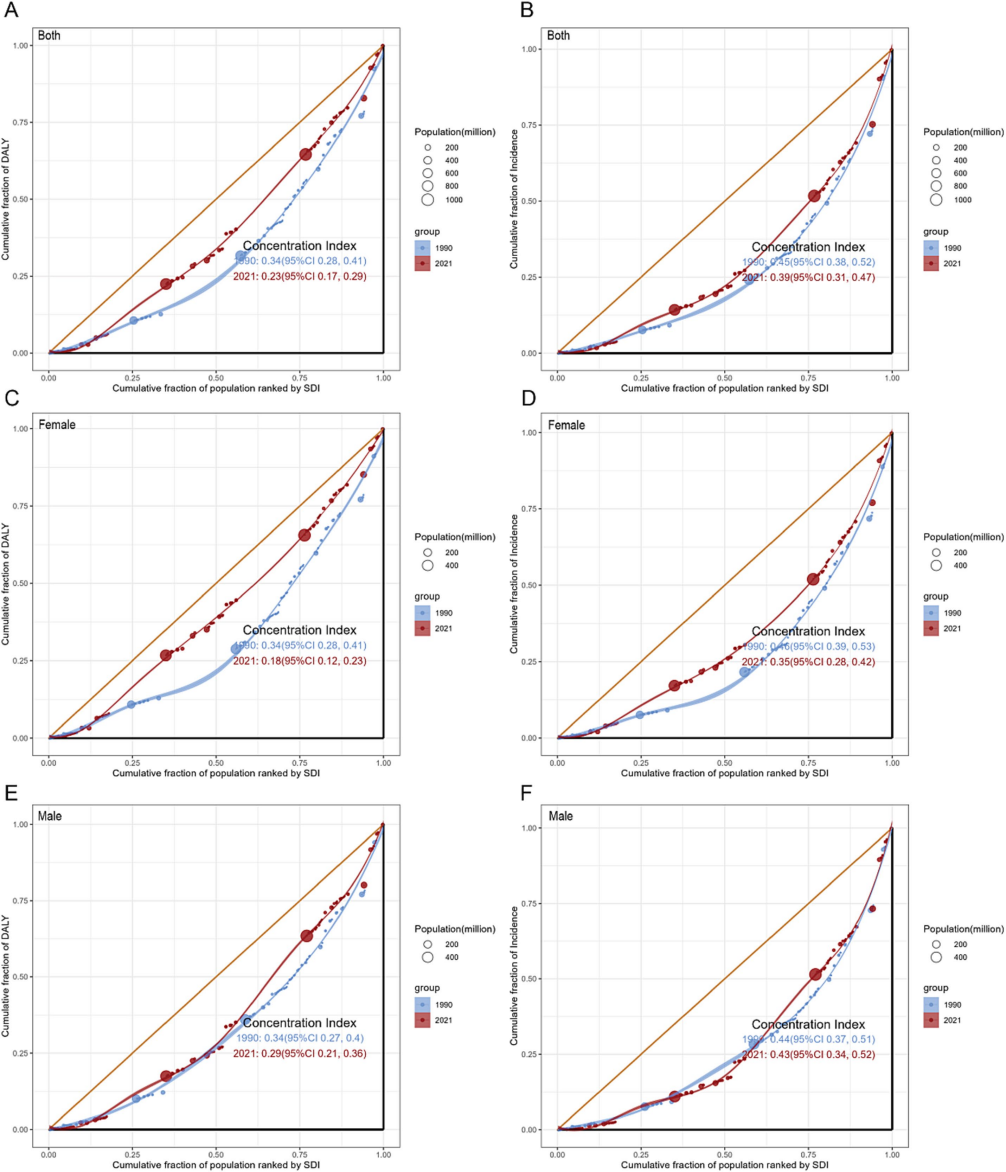
Concentration curves for GBTC worldwide in 1990 and 2021: DALYs by whole population **(A)**, Females **(C)**, and Males **(E)**; incidence by whole population **(B)**, Females **(D)**, and Males **(F)**. CI, concentration index; DALYs, disability-adjusted life-years; GBTC, gallbladder and biliary tract cancer.

The concentration index (CI) (Figure 5) for total population DALYs demonstrated a significant decline from 0.34 (95% CI: 0.28 to 0.41) in 1990 to 0.23 (95% CI: 0.17 to 0.29) in 2021 (pval = 0.009). Conversely, the reduction in the CI for incidence from 0.45 (95% CI: 0.38 to 0.52) in 1990 to 0.39 (95% CI: 0.31 to 0.47) in 2021 was not statistically significant (pval = 0.228). Among females, significant decreases in CI were observed for both DALYs and incidence from 1990 to 2021 (pval < 0.05), while in males, decreases were noted but did not reach statistical significance (pval > 0.05). Overall, the observed reduction in cross-country disparities in DALYs within the total population was primarily attributed to improvements among females. Additionally, when analyzed according to the SDI (Supplementary Table S6), the low SDI quintile demonstrated an increasing CI for both measures. This trend, alongside the previously noted decrease in the SII, indicates a progressive advancement in the management of GBTC, albeit with a more uneven distribution.

### Decomposition analysis

**Figure 6:**
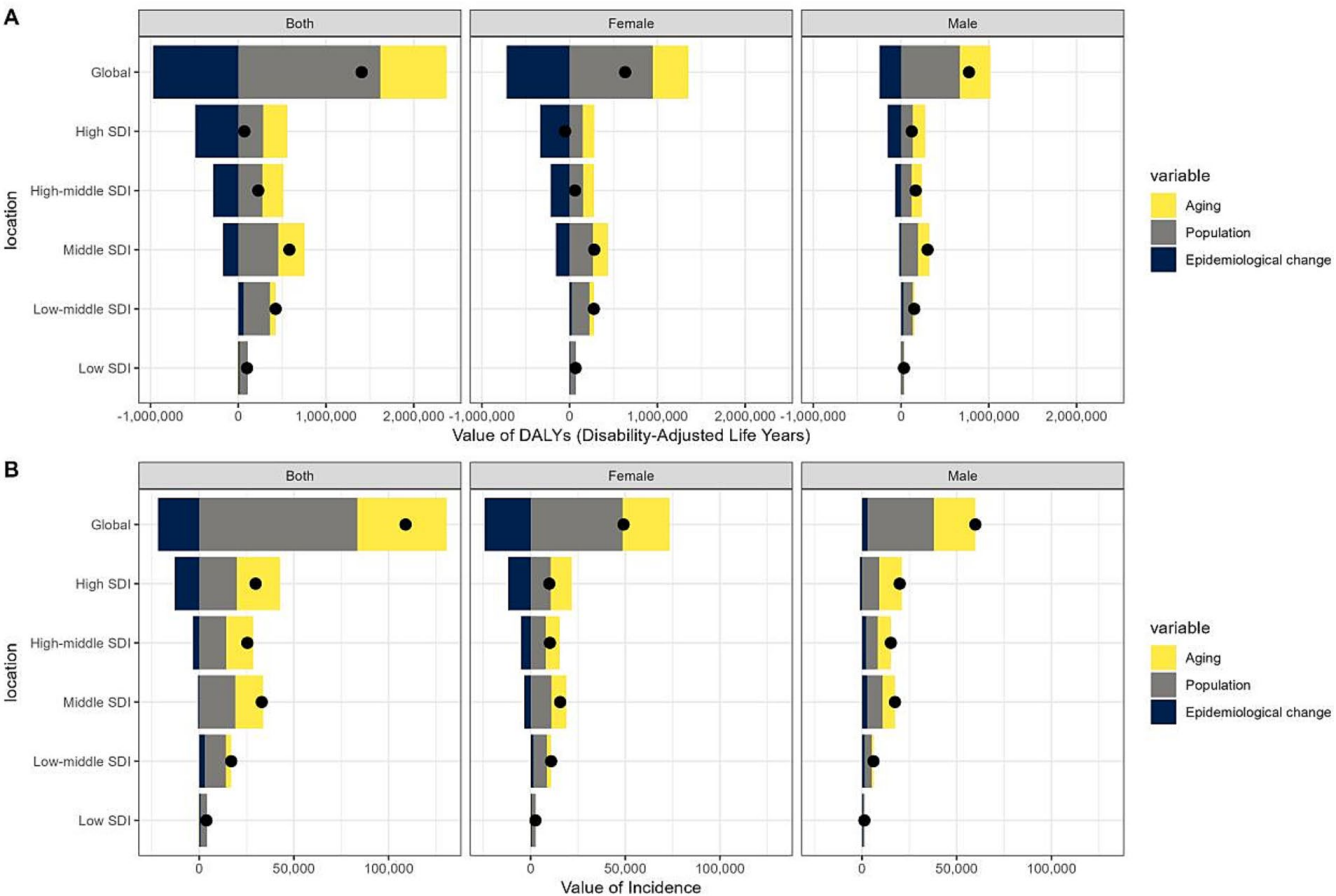
Changes in DALYs **(A)** and incidences **(B)** of gallbladder and biliary tract cancer (GBTC) globally and by SDI quintile. A positive magnitude indicates an increase in numbers attributable to the component, a negative magnitude indicates a decrease in attribution, and a black point represents the overall number. GBTC, gallbladder and biliary tract cancer; SDI, socio-demographic index; DALYs, disability-adjusted life-years.

Over the past 31-years, there has been a significant rise in GBTC DALYs, most pronounced in the middle SDI quintile, with the high SDI quintile showing the smallest increase (Figure 6A, Supplementary Table S7). From 1990 to 2021, the global increase in GBTC DALYs was mainly due to population growth (115.05%), followed by ageing (53.86%), counteracted by an epidemiological change-induced reduction of −68.91%. The high SDI quintile experienced the greatest impact of population growth on DALYs (410.27%), whereas the low-middle SDI quintile had the least impact (71.07%). The effect of population ageing on DALYs generally increased across most regions, with a slight decrease in the low SDI quintile (−6.38%). Regarding epidemiological changes, a widespread decrease in DALYs was observed globally across different SDI quintiles, most notably in the high SDI quintile (−705.84%), which mitigated the increasing trend due to population structure changes. Conversely, the low and low-middle SDI quintiles continued to present an upward trend attributed to epidemiological change, with increases of 12.37 and 13.75%, respectively.

The global increase in GBTC incidence (Figure 6B, Supplementary Table S8) was primarily due to population growth (76.67%), with ageing (43.26%) also playing a significant role, partially offset by a reduction of −19.92% from epidemiological changes. However, the distribution of incidence increases across SDI quintiles differed from that of DALYs, with the majority of the global rise occurring in higher quintiles. The impact of population growth intensified globally and was the predominant factor in the change of the low SDI quintile (87.68%). The contributions of population ageing and epidemiological changes to the overall incidence in different SDI quintiles followed a similar pattern to that of DALYs. Notably, in males, epidemiological changes contributed 4.87% to the global incidence change, showing an overall increase across all SDI quintiles, except for a slight decrease in the high SDI quintile (−5.56%).

## Discussion

Although surgery is currently the sole curative treatment for GBTC, diagnosis (27) often occurs at an advanced stage, limiting its feasibility. Surgical complexities (28) and high complication rates further impede effective treatment. Conventional chemotherapies, such as Cisplatin-Gemcitabine, remain the backbone of first-line treatment (29), while recent advances in targeted therapies have provided new options for patients with actionable molecular alterations (30), including target-inhibitors against EGFR, IDH1, and NTRK (31). Moreover, immunotherapy (32), such as Pembrolizumab for MSI-H tumors, has expanded the therapeutic landscape. Despite attempts to address these issues, recurrence rates for GBTC remained high, highlighting the ongoing burden of this disease (33). These advancements in medical care have led to a significant decline in the overall ASDR and ASIR across the three decades on the global scale. However, variations in trends were observed across different SDI quintiles. In higher SDI regions, the accessibility to timely detection, extensive use of abdominal ultrasound, broader coverage of healthy insurances, and progress on precise therapies significantly alleviated the overall incidence and DALYs of GBTC. However, the regions with lower SDI still suffered from increasing disease burden. Specifically, for DALYs, low-middle SDI quintiles have risen to the highest in 2021.

**Figure 7:**
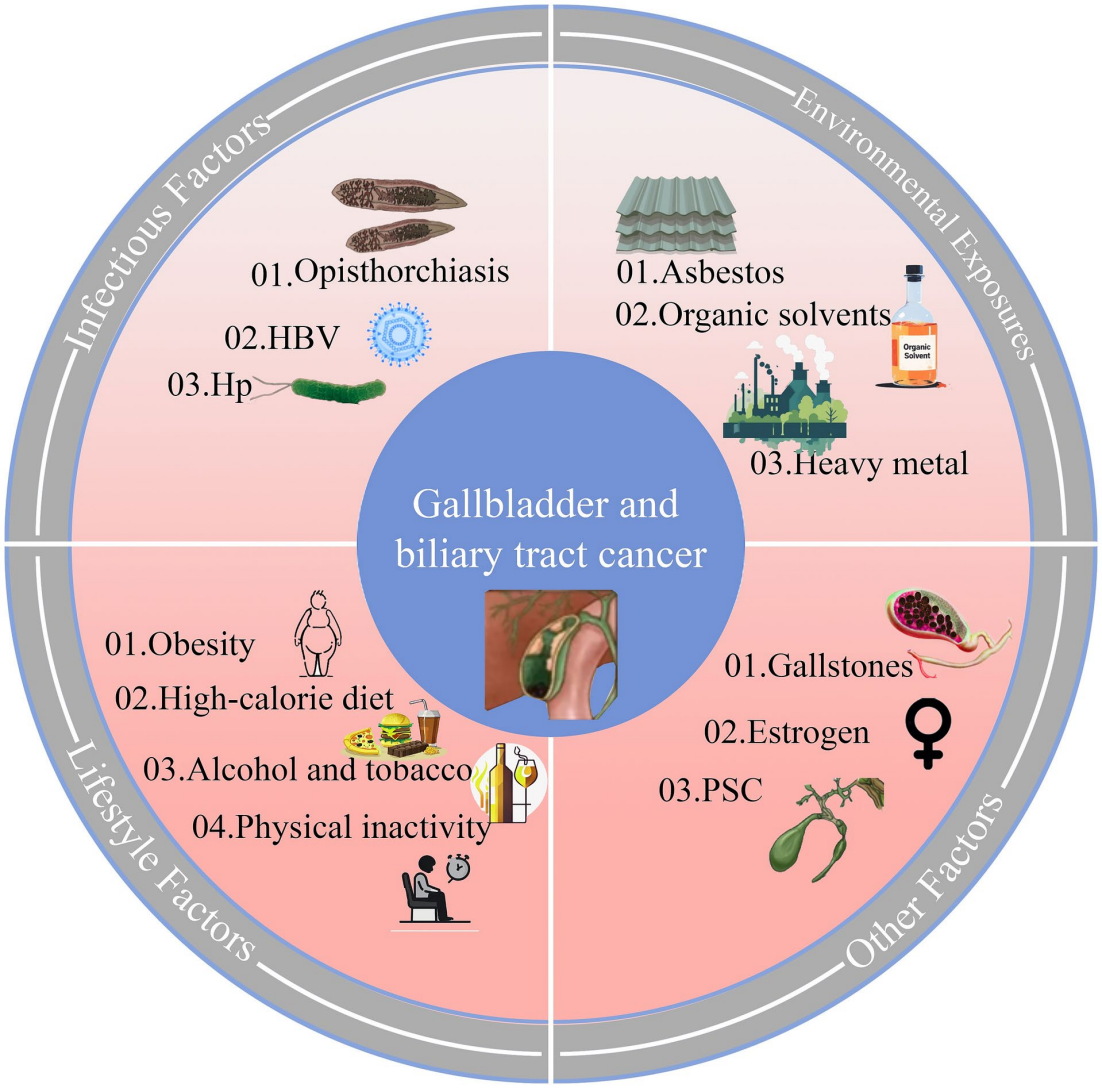
Biological pathways and risk factors in Gallbladder and Biliary Tract Cancer (GBTC). HBV, hepatitis B virus; Hp, *Helicobacter pylori*; PSC, Primary sclerosing cholangitis.

As illustrated in Figure 7, on the occurrence of GBTC, Primary sclerosing cholangitis (PSC) (34) was the main reason in Europe and North America, and asbestos was responsible for that in Western countries (35). Liver fluke infection in Southeast Asia (36), Hepatitis B virus in China (37), and organic solvents (1,2dichloropropane) in Japan (38) were also confirmed to induce GBTC. Environmental factors, economic development stages, and local etiologies influence geographical disparities. For instance, in Chile, high incidences and DALYs were thought to be linked to imbalanced educational levels, a high rate of obesity, and a high prevalence of cholelithiasis (39). Furthermore, as a major global copper producer since 1985, Chile has faced heavy metal contamination and wastewater issues. This has led to implementing policies aimed at limiting the release of pollutants into marine and freshwater bodies (40) and establishing specific programs to enable early detection and treatment for high-risk groups with gallbladder and biliary tract cancer (12). Our study shows that despite its heavy basic disease burden, Chile’s achievement against GBTC in recent decades is notable and offers valuable lessons.

Gender differences in incidence and DALYs of GBTC were evident both globally and across different SDI quintiles, showing a reversal trend and highlighting the need for targeted interventions. It is widely recognized that females are more susceptible to gallbladder cancer than males (41), especially in lower SDI regions. Prior research has shown that estrogen-induced cholestasis is a common liver disease (42) that can lead to gallstone-mediated gallbladder cancer (43). Moreover, estrogen binds to estrogen receptors (ESR) in various tissues, including the biliary tract (44), which may influence the function of the biliary tract system. In lower SDI regions, unequal social status, more crude productive methods, unsanitary diets, and a shortage of medical support may exacerbate females` burden of GBTC. On the other hand, early fertility and multiparity were associated with gallstones (45), which were considered the main reasons for GBTC. Based on the high burden of early years, DALYs of females presented a much more drastic decline than males globally in recent years but were still higher than males in most regions. In low- and low-middle SDI quintiles, the burden of GBTC, measured by both DALYs and the incidence for both genders, continued to rise, indicating a need for increased assistance for countries lagging.

Notably, unexpected reversal trends were observed in higher SDI quintiles, where both the DALYs and incidence of males have been surpassing those of females in recent years, with the gap projected to increase. Advances in detection techniques partially explain the global increase in incidence, but the divergent trends between males and females are unusual. The same phenomenon was observed in DALYs, which is concerning. General risk factors associated with higher living standards, such as type 2 diabetes, alcohol and tobacco use, and high energy food consumption, contribute to the increasing incidence of cholangiocarcinoma (46). Reduced physical activity has also been associated with the risk of major hepatobiliary cancers (47), gallstones and gallbladder cancer (48). These factors may contribute to the rising burden among males in higher SDI quintiles and explain why the burden of GBTC is also increasing in lower SDI quintiles.

Despite improvements in medical technology and healthcare delivery, our study reveals that the burden of GBTC remains disproportionately higher in high SDI regions compared to lower SDI areas. This finding is counterintuitive, as high SDI regions typically have better healthcare systems and resources. However, several factors may contribute to this unexpected result. High SDI regions often experience more pronounced population aging, and GBTC incidence increases with age. Additionally, these regions may face higher levels of industrial pollution and environmental exposure, which could contribute to the higher burden. Furthermore, the stronger detection and diagnostic capabilities in high SDI regions may lead to earlier and more accurate identification of GBTC cases, resulting in higher reported incidence rates and DALYs.

Regarding the unexpected reversal of gender differences, our study found that while females traditionally had higher DALYs and incidence rates, recent trends show that males in high SDI regions are now experiencing higher rates. This reversal may be attributed to significant lifestyle changes among males, such as increased obesity, alcohol and tobacco use, and reduced physical activity. In contrast, improved health awareness and better access to medical care among females may have contributed to a decline in their DALYs and incidence rates. These findings highlight the complexity of GBTC and the need for further research to better understand the underlying factors contributing to these trends. Targeted interventions and policies addressing environmental exposures, lifestyle factors, and gender-specific risks are necessary to effectively reduce the burden of GBTC in high SDI regions.

The joinpoint regression analysis identified significant changes in the trends of ASIR and ASDR over the study period. For females, both ASDR and ASIR continuously decreased, with AAPCs of −1.27% (95% CI: −1.34 to −1.20) and −0.82% (95% CI: −0.94 to −0.70), respectively. In contrast, while ASDR for males also decreased (AAPC = −0.53%; 95% CI: −0.63 to −0.43), the ASIR increased (AAPC = 0.23%; 95% CI: 0.08 to 0.38). These findings indicate a significant gender disparity in the burden of GBTC, with males experiencing an increasing ASIR despite a decreasing ASDR. While the BAPC model provides a robust framework for projecting future disease burden, it is important to note that projections are based on the continuation of historical trends. Sudden policy changes, advancements in medical technology, or significant shifts in resource allocation could alter the projected outcomes. Additionally, the model assumes that the data conforms to a Poisson distribution, which may introduce some bias in the projections.

Inequality analysis underscored substantial disparities, with higher SDI countries shouldering a disproportionate burden. The SII and CI only demonstrated a slight gap from 1990 to 2021. Despite the theoretical expectation that regions with higher SDI would exhibit better healthcare quality and higher health standards, our analysis revealed a counterintuitive observation of a higher burden of GBTC in higher SDI areas. Several factors may contribute to this unexpected outcome. The correlation between social development and increased industrial pollution and environmental exposure suggested a potential link (49), with environmentally induced genetic mutations potentially playing a critical role in the initiation of cancer development (50). Besides, countries with higher SDI boasted larger population bases and experienced more pronounced ageing trends, coupled with advanced and widespread medical services, contributing to higher GBTC detection rates. Furthermore, the high quality of life in these regions may contribute to obesity (51), a well-recognized factor in gallstone disease, thereby increasing the risk of GBTC. Despite the advancements in treatment, the elusive nature and poor prognosis of GBTC (52) persist, with long-term survival rates remaining low (53). Although concerted efforts in high SDI regions had been made to alleviate the GBTC burden, as indicated by this analysis, there remained a substantial journey ahead in comprehensively addressing and reducing the impact of GBTC on global health. Moreover, the development of precision medicine inevitably relies on the overall regional level of development. Conducting clinical trials free of charge in less developed regions and medical support from affluent areas may yield additional clues and extend benefits to a broader population. Our study observed that the primary promotion of narrowing health inequalities was driven by females, while inequalities among males remained stable globally and worsened regionally. Despite the common understanding that the female gender is a risk factor for biliary diseases, previous policies focusing on females may have overlooked the conditions of males.

Decomposition analysis highlighted the intricate relationship between population dynamics and epidemiological factors in shaping GBTC burden. Population growth emerged as the primary driver contributing to the DALYs increase, followed by ageing effects. Epidemiological changes exhibited a global decrease, with the high SDI quintile experiencing the most significant reduction. Our decomposition analysis further revealed that population growth was the dominant driver globally, accounting for 115.05% of DALY increases and 76.67% of incidence increases, with particularly strong effects in high SDI regions (410.27% for DALYs). Population aging contributed an additional 53.86% of global DALY increases and 43.26% of incidence increases, showing growing impacts across most regions except the low SDI quintile. These demographic factors were partially offset by epidemiological changes, which showed a global reduction (−68.91% for DALYs, −19.92% for incidence), though with significant regional variation. High SDI regions demonstrated substantial epidemiological improvements (−705.84% for DALYs), reflecting successful prevention and treatment efforts, while low and low-middle SDI regions showed concerning worsening trends (+12.37–13.75%). With the global demographic shift towards an ageing population, the current significant challenges of ageing and cancer are expected to escalate into larger public health and socioeconomic issues (54). Population growth and ageing are inevitably associated with the burden of GBTC and were hard to eliminate, but other risk factors could still be tackled. GBTC results from a variety of factors (55), including chronic inflammation (56) origin from lithiasis or non-lithiasis, *Helicobacter pylori* infection, obesity (57), environmental exposure, and infectious parasites. Characterized by the poor prognosis of GBTC, effective interventions focusing on its onset or delaying the progression apply to all age stages (58), which also explains the anti-DALYs contribution of epidemiological changes in regions with higher quintiles. Regarding incidence rates, most of the increase was observed in higher SDI quintiles, which is consistent with our previous findings. The analysis also revealed important gender differences, with males benefiting less from epidemiological improvements than females. These findings explain several key patterns: the global decrease in ASDR despite rising case numbers (where demographic growth was outweighed by epidemiological improvements), the persistent burden in high SDI regions (due to aging populations despite strong epidemiological progress), and the growing challenges in low SDI regions (facing both demographic pressures and worsening epidemiological trends). The results underscore the need for differentiated policy approaches - sustained investment in prevention and treatment for aging populations in high SDI regions, urgent attention to basic healthcare infrastructure in low SDI regions, and gender-specific strategies given males’ slower progress in epidemiological improvements.

The study presents several limitations. Firstly, heterogeneity in GBTC characteristics and data collection methods across countries introduces uncertainty in global estimates of GBTC DALYs. This variability may impact the accuracy and comparability of the study results. Secondly, relying solely on the SDI as an index to represent overall country development may oversimplify the complex factors influencing the GBTC burden, such as lifestyle and environmental factors. Moreover, our BAPC model did not account for the impacts of sudden policy changes or significant shifts in resource allocation, and we could not project SDI quintiles further due to the dynamic nature of SDI values. Additionally, using joinpoint regression based on modeled GBD data rather than original raw data may have introduced some inherent bias. Future research could benefit from analyzing extended datasets to comprehensively understand GBTC trends. It is important to note that the GBD study is an ongoing and evolving project, and the data used in this analysis cover the period from 1990 to 2021. Subsequent updates to the GBD dataset may refine the estimates and provide additional insights.

## Conclusion

Our study highlights significant disparities in the global burden of GBTC, with higher burdens in high SDI regions and notable gender differences. These trends are likely influenced by population aging, environmental exposures, and lifestyle changes such as increased obesity and reduced physical activity. However, our analysis is limited by data heterogeneity and reliance on the SDI as a developmental indicator. Future research should focus on extended datasets and region-specific analyses to better understand the impact of environmental and lifestyle factors on GBTC. Policymakers should prioritize resource allocation for early detection programs and healthcare infrastructure, especially in low-SDI regions. International collaboration is essential for sharing best practices and reducing global inequalities in GBTC management.

## Data Availability

Publicly available datasets were analyzed in this study. This data can be found here: http://ghdx.health data.org/gbd-results-tool.
