# Supplementary material for: Global burden and cross-country inequalities in gallbladder and biliary tract cancer (1990–2021) with projections to 2050: insights from the global burden of disease study 2021
Source: Front Med (Lausanne). 2025 May 12;12:1520714. doi: 10.3389/fmed.2025.1520714 (PMC12104178; doi:10.3389/fmed.2025.1520714)
Supplement: Supplementary file 1 [file Data_Sheet_1.docx]

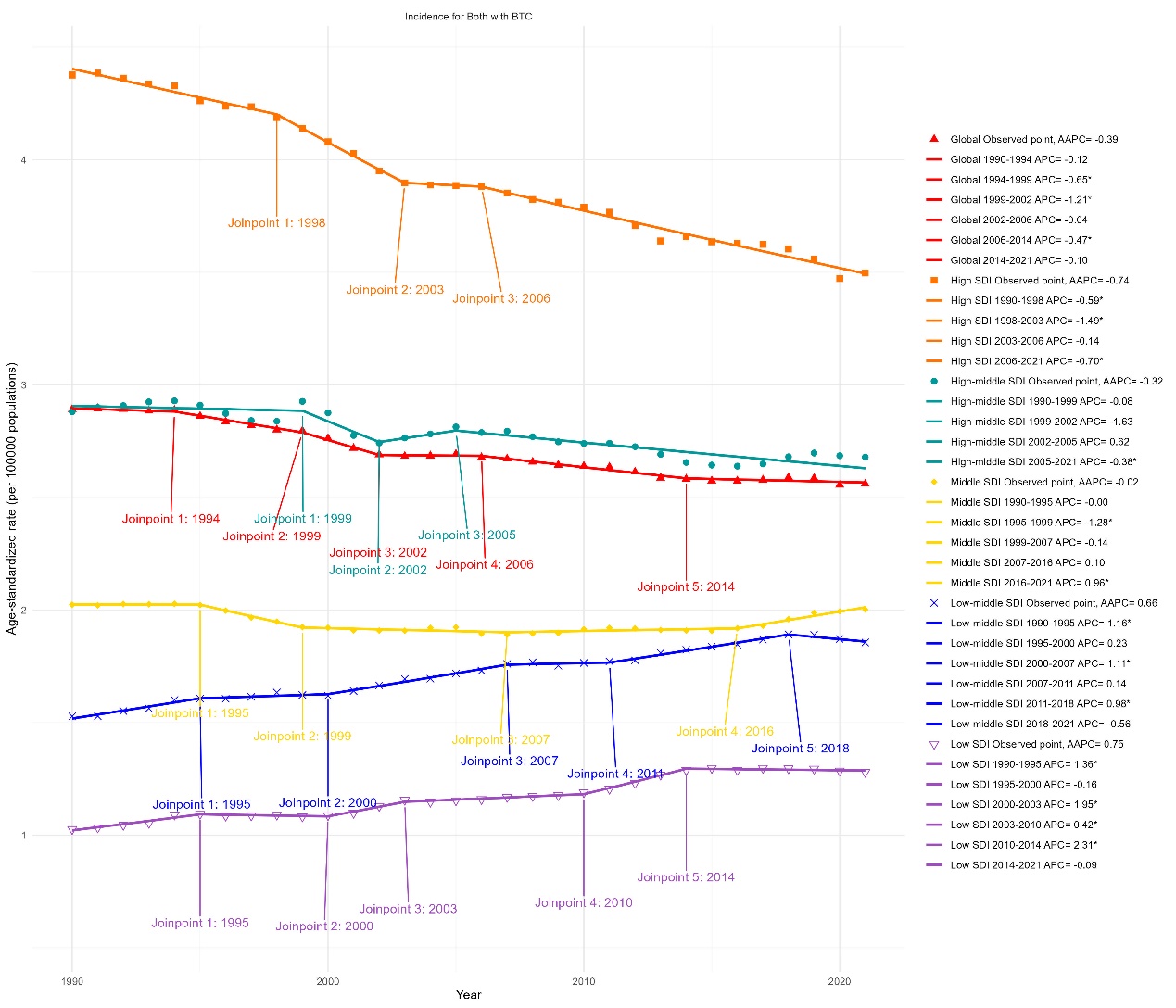


**Figure S1. Global trends of ASDR of GBTC stratified by SDI quintiles from 1990 to 2021.**

ASDR, age standardized disability-adjusted life-years rate; GBTC, gallbladder and biliary tract cancer; AAPC, average annual percent change; APC, annual percent change; SDI, Socio-Demographic Index.


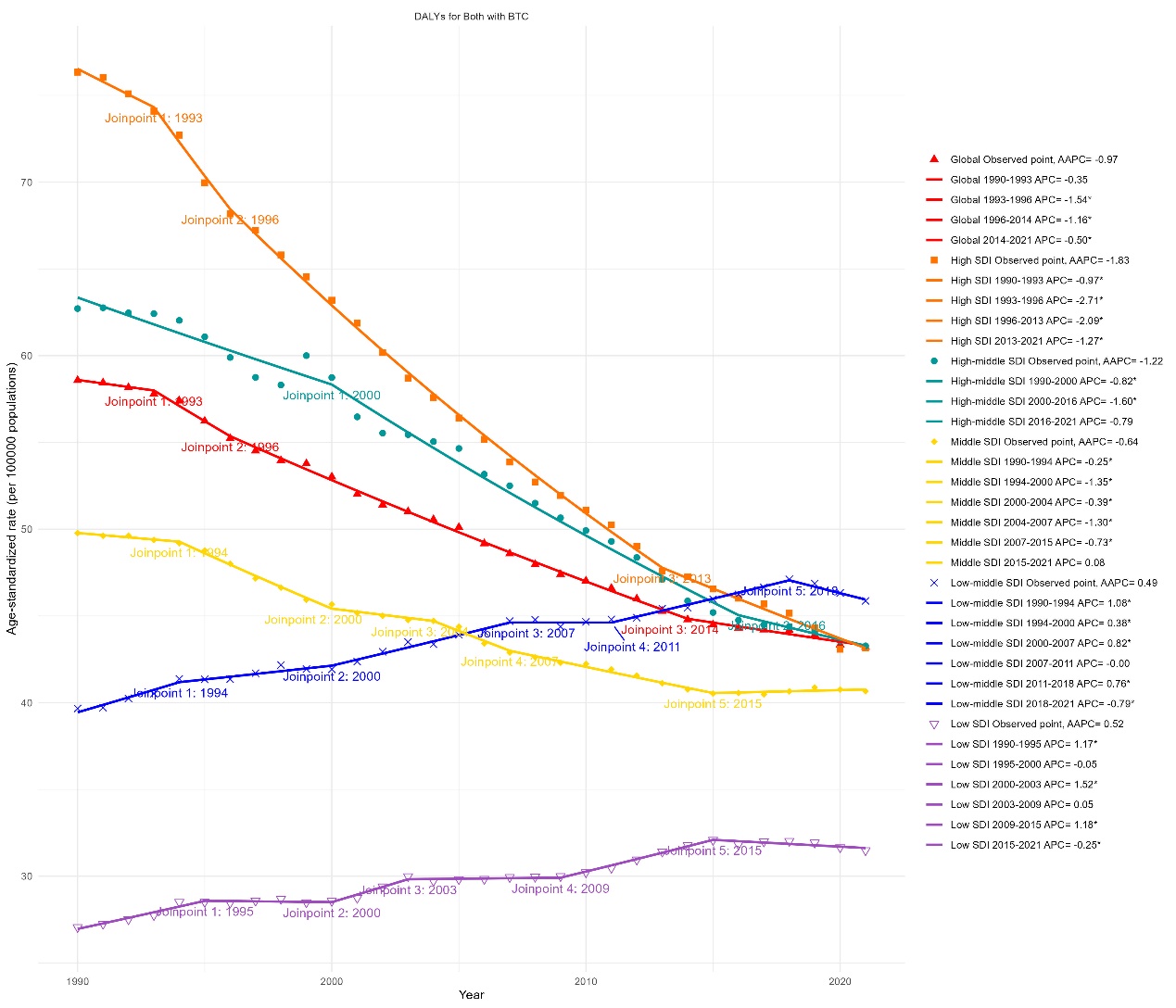


**Figure S2. Global trends of ASIR of GBTC stratified by SDI quintiles from 1990 to 2021.**

ASIR, age standardized incidence rate; GBTC, gallbladder and biliary tract cancer; AAPC, average annual percent change; APC, annual percent change; SDI, Socio-Demographic Index.


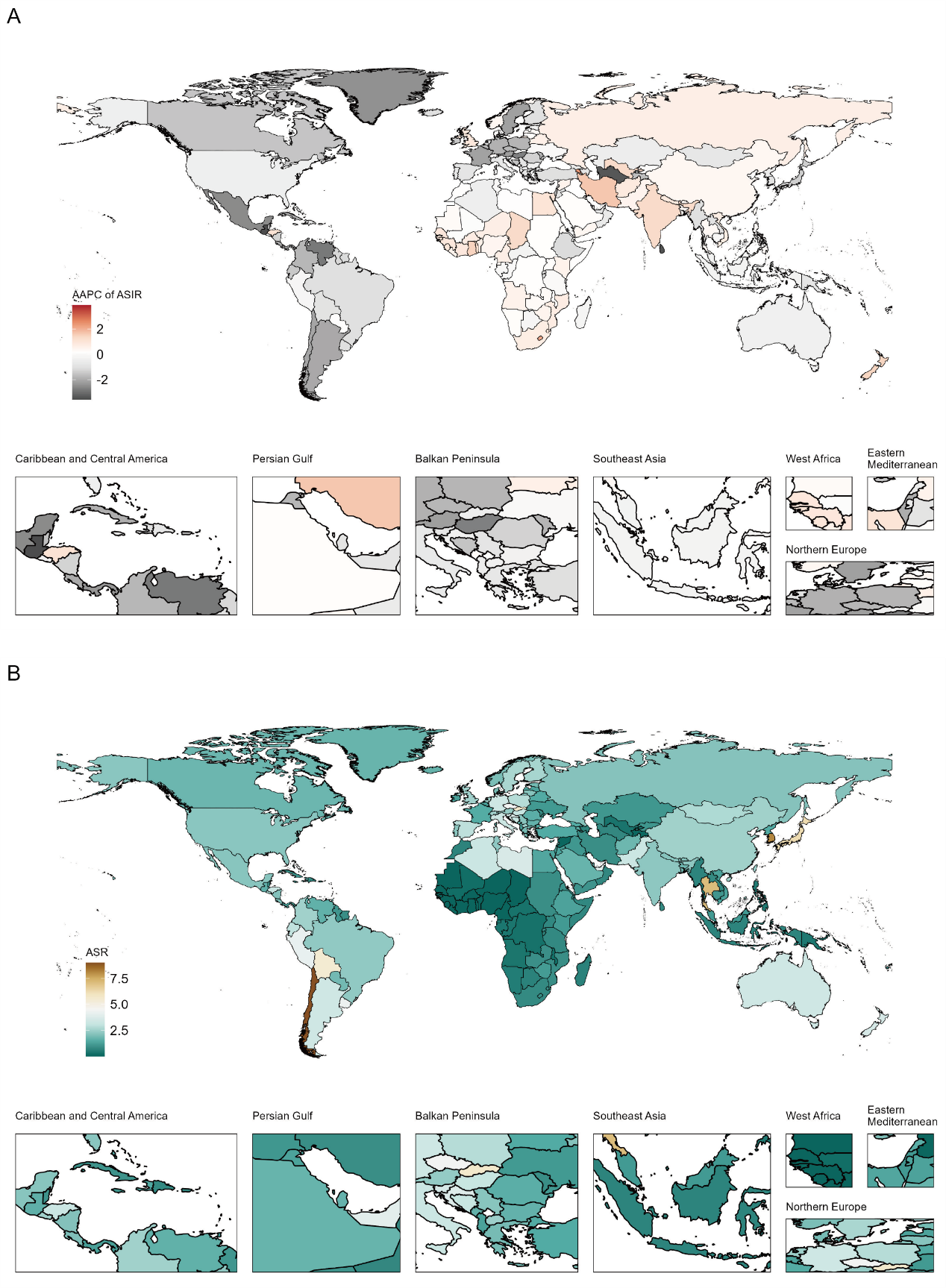


**Figure S3. Average Annual Percent Change (AAPC) in ASIR of GBTC from 1990 to 2021 (A), and ASIR values for GBTC in 204 countries and territories in 2021 (B).**

ASIR, age standardized incidence rate; GBTC, gallbladder and biliary tract cancer; AAPC, average annual percent change.


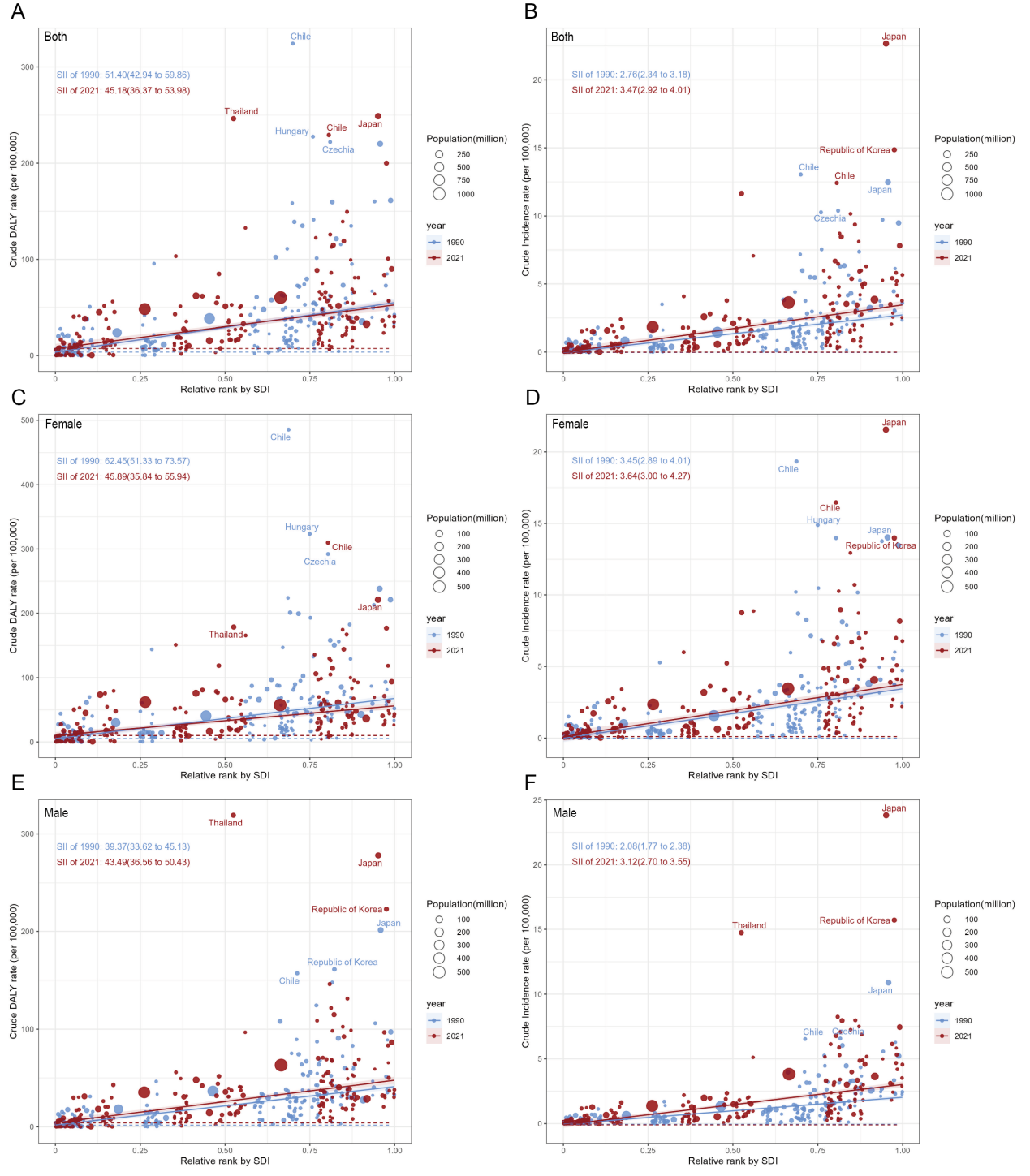


**Figure S4. Health inequality regression curves for the DALYs and incidence of GBTC worldwide and by genders, in 1990 and 2021.**

SII, slope index of inequality; DALYs, disability-adjusted life-years; GBTC, gallbladder and biliary tract cancer.
